# Evaluation of the characteristics of older patients admitted to a general ICU

**DOI:** 10.1186/cc14679

**Published:** 2015-09-28

**Authors:** Bruno F Mazza, Ana Lúcia V Ronchini, Dario F Ferreira, Joely L Malachia, Rosa GA Rocha, Samantha L Almeida

**Affiliations:** 1 Hospital Samaritano, Higienópolis, São Paulo, São Paulo, Brazil

## Introduction

Aging is a fact that occurs today, and is associated with increased prevalence of chronic diseases and functional impairment. This leads to an increase in hospitalization, especially in ICUs. A better understanding of the characteristics of these patients is essential to provide the best assistance we can and to have the best of the resources needed for the proper treatment of these patients [1].

## Objective

The aim of this study is to compare the epidemiological characteristics of older patients (>70 years) with those with lower age (<70 years), admitted to a general ICU.

## Methods

A retrospective analysis was performed from June to December 2014, using the database EPIMED^®^. We evaluated 758 patients who were hospitalized in two ICUs, corresponding to 37 beds. Statistical analysis was performed using SPSS 22, using the Student *t *test for numerical variables and the chi-square test for categorical variables. We considered statistically significant a *p *value <p.05.

## Results

Patients older than 70 years accounted for 50% of admissions; 68% of clinical admissions and 32% surgical hospitalizations-when compared for the group <70 years, we have 60% of clinical admissions and 40% surgical (*p *= 0.15), with 50% of these coming from the emergency room. There was no difference between groups in length of stay in the ICU (>70: 6.73 ± 7.76 vs. 70: 12.0 ± 22.3 vs. < 70: 244.3 ± 18.38) (*p *= NS). The group >70 years, however, showed a higher value of SAPS 3 (55.6 ± 15.12 vs. 41.6 ± 16.2, *p *= 0.01) and a greater likelihood of death (39% vs. 19.5%). Group > 70 years was higher (13% vs. 8%, *p *= 0.01); however, the standardized mortality rate (SMR) for the group >70 years was 0.49 and for the group <70 was 0.45.

## Conclusion

In our study, we found a high hospitalization rate in patients older than 70 years in the unit, despite a higher SAPS 3, and no difference in length of stay in the ICU or hospital was shown. The group >70 years had a higher mortality but the adjusted mortality rate shows a good performance in both groups of patients. Increasing age is a fact and it is important be prepared to manage this kind of patient to give them the best possible care.
